# Supplementary figures and images for: Learning to perform role-filler binding with schematic knowledge
Source: PeerJ. 2021 Mar 31;9:e11046. doi: 10.7717/peerj.11046 (PMC8019313; doi:10.7717/peerj.11046)

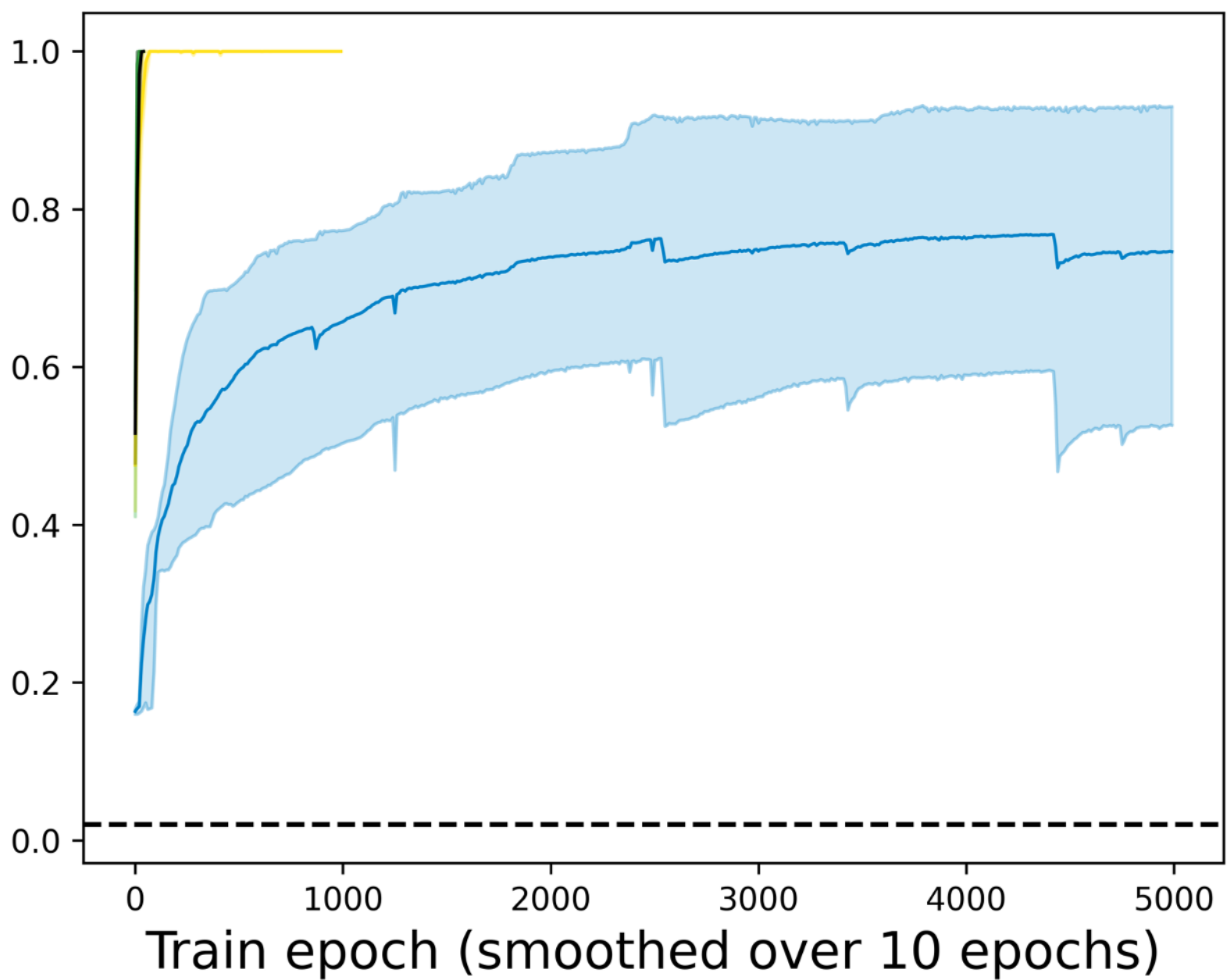

A

— RNN    — LSTM    — Fast Weights    — DNC    --- Chance Rate

B

Supplement: Supplemental Information 1 [file peerj-09-11046-s001.pdf]

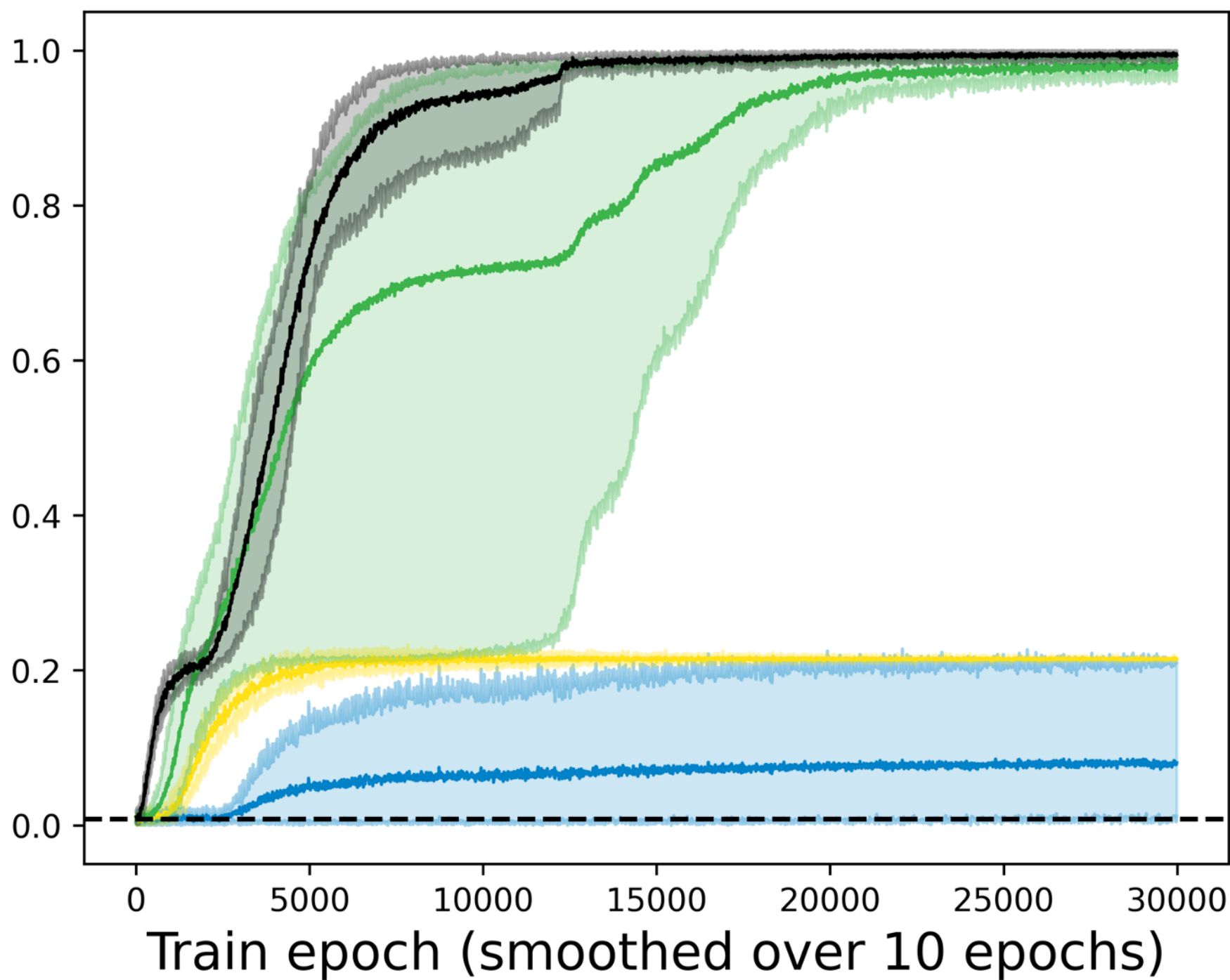

A

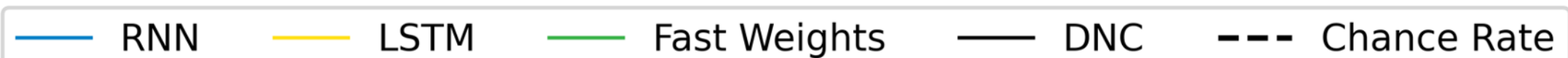

B

Supplement: Supplemental Information 2 [file peerj-09-11046-s002.pdf]

Histogram of Write Weights by Filler

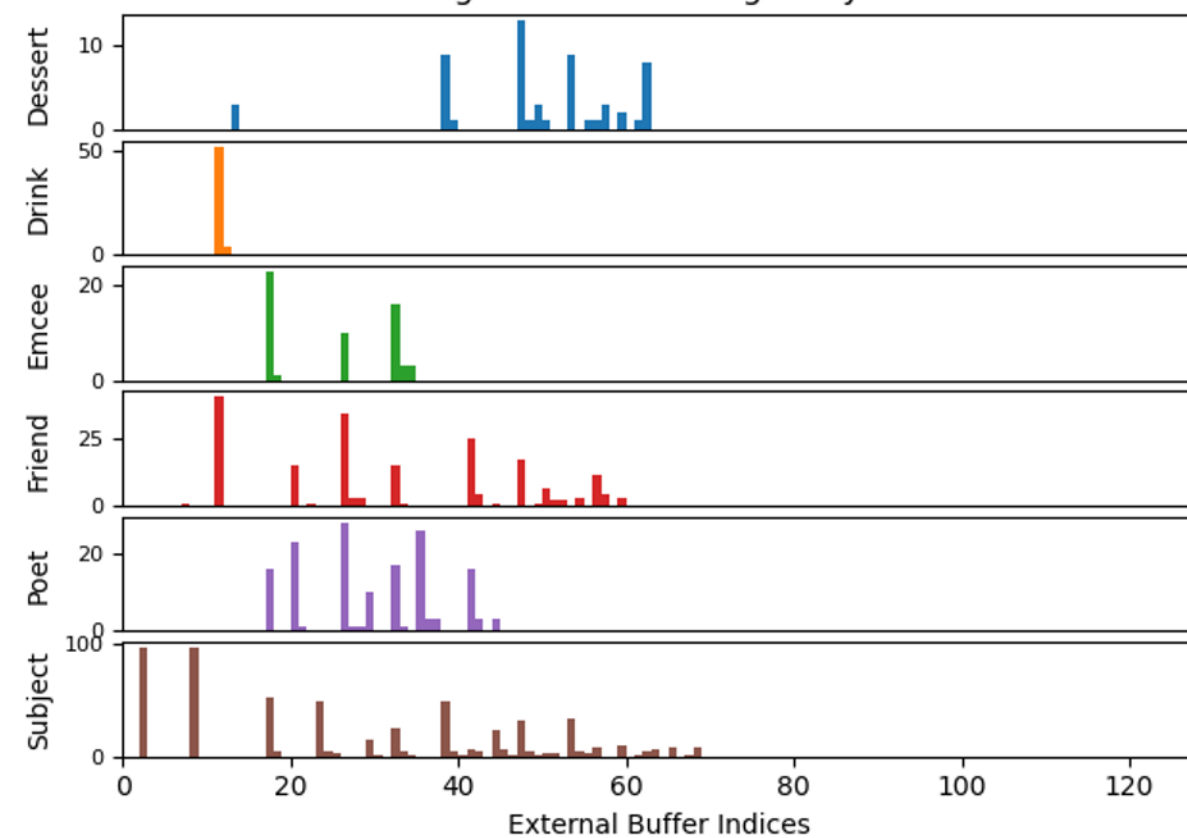

Histogram of Read Weights by Query

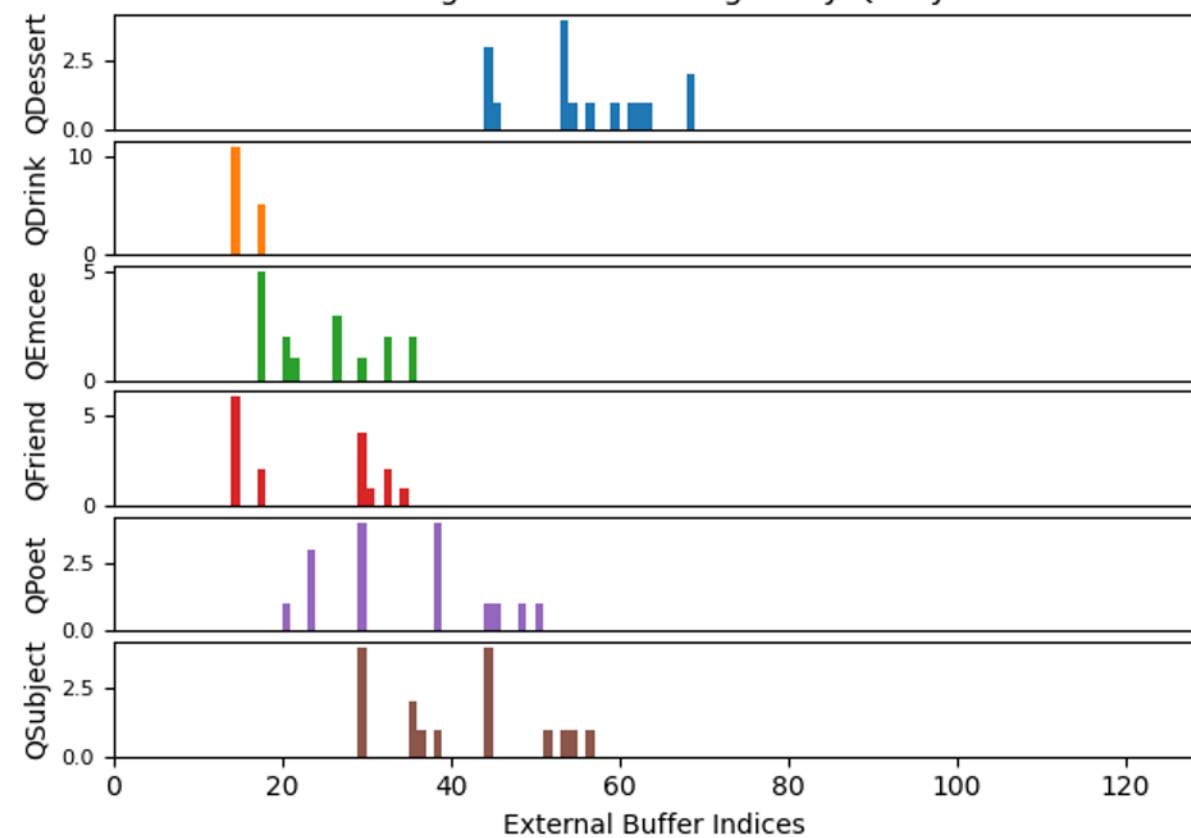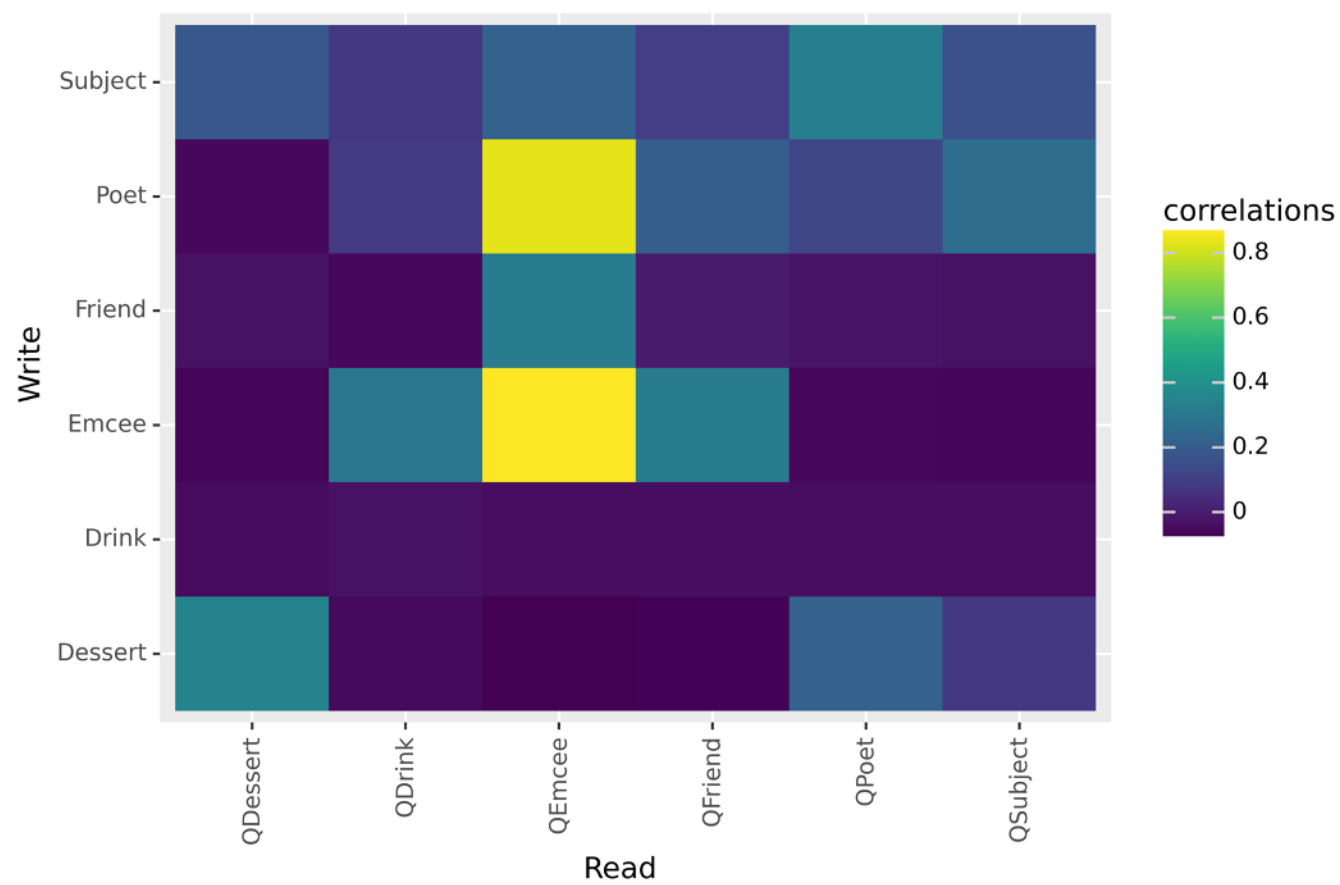

Supplement: Supplemental Information 3 [file peerj-09-11046-s003.pdf]

Histogram of Write Weights by Filler

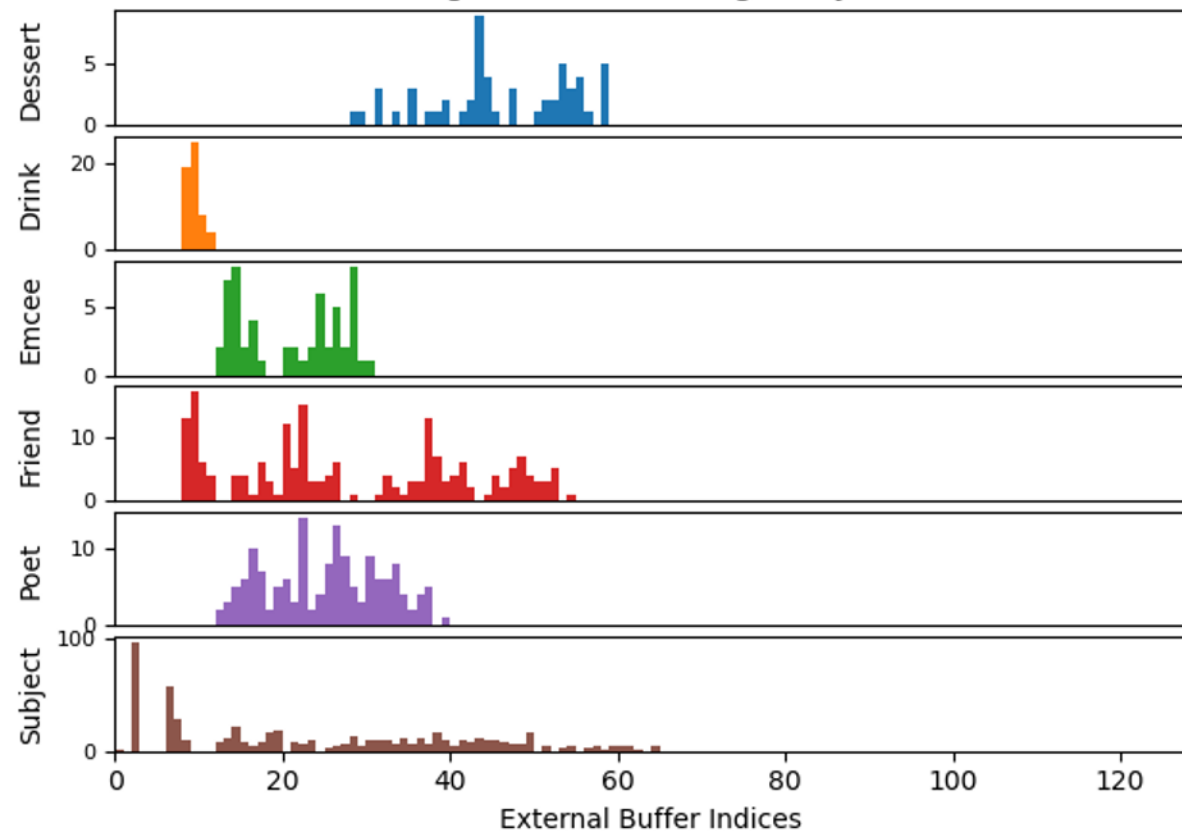

Histogram of Read Weights by Query

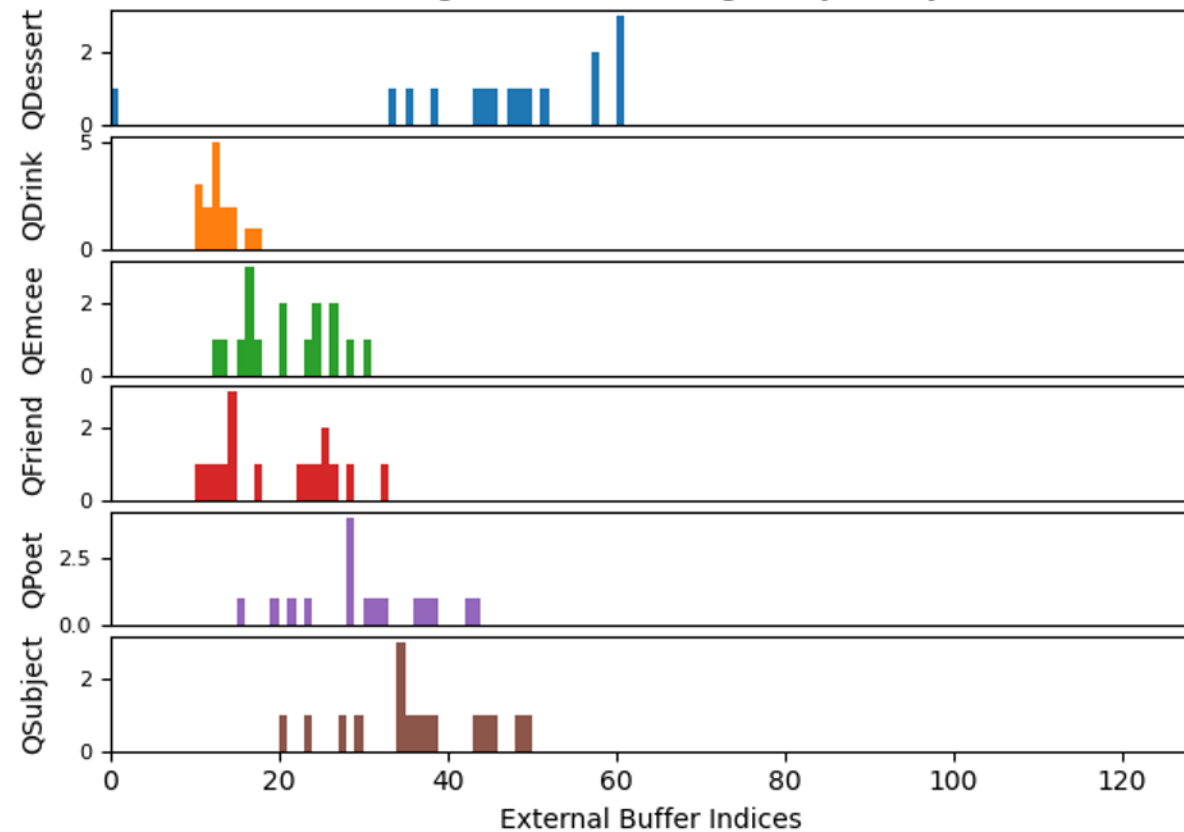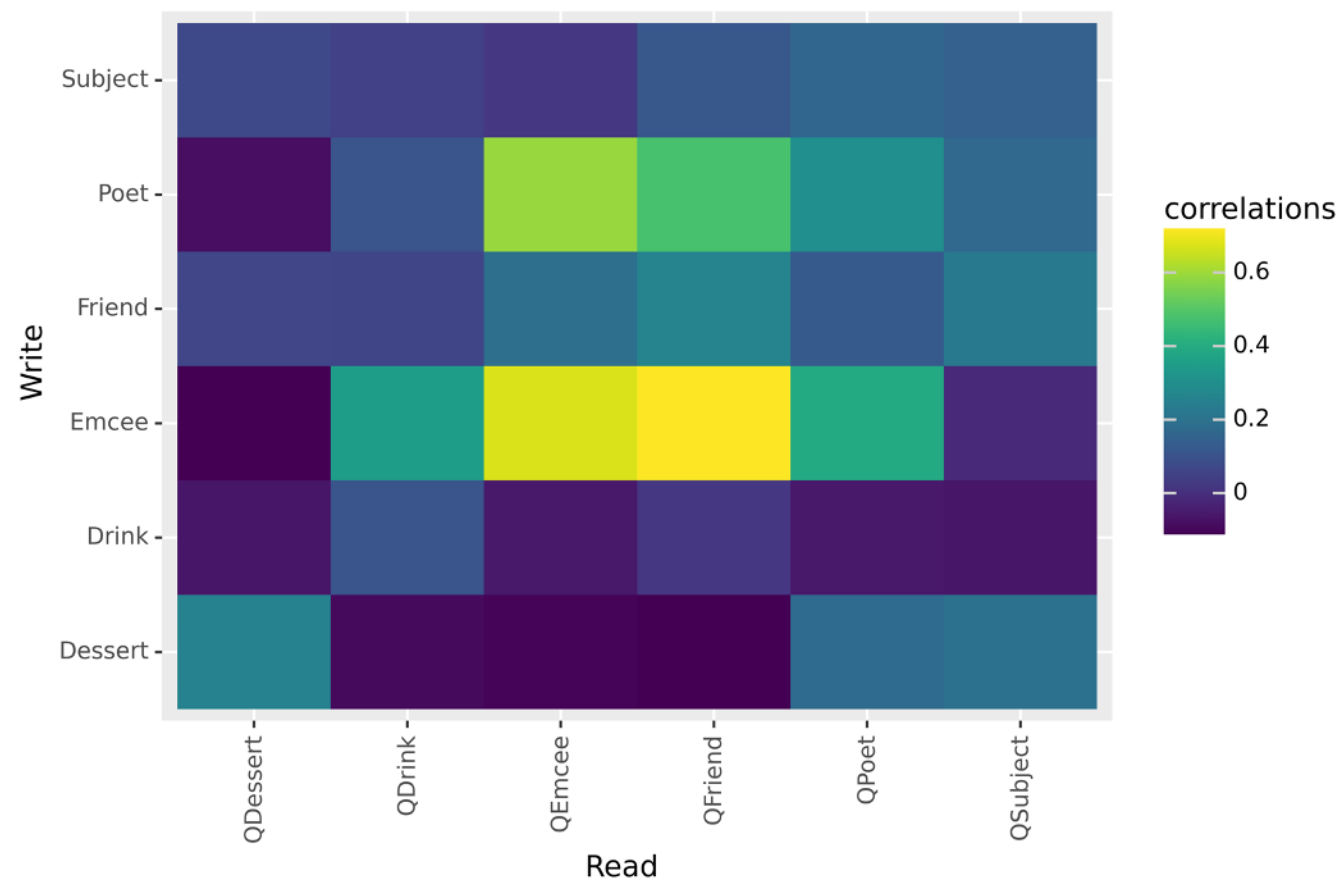

Supplement: Supplemental Information 4 [file peerj-09-11046-s004.pdf]

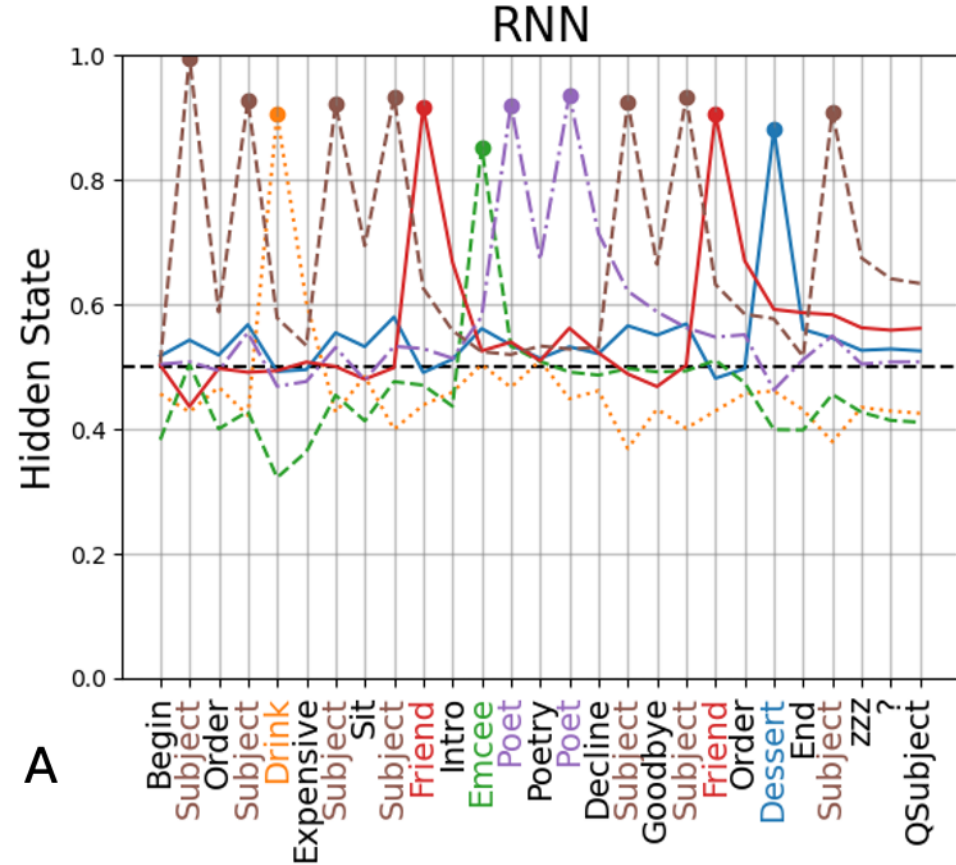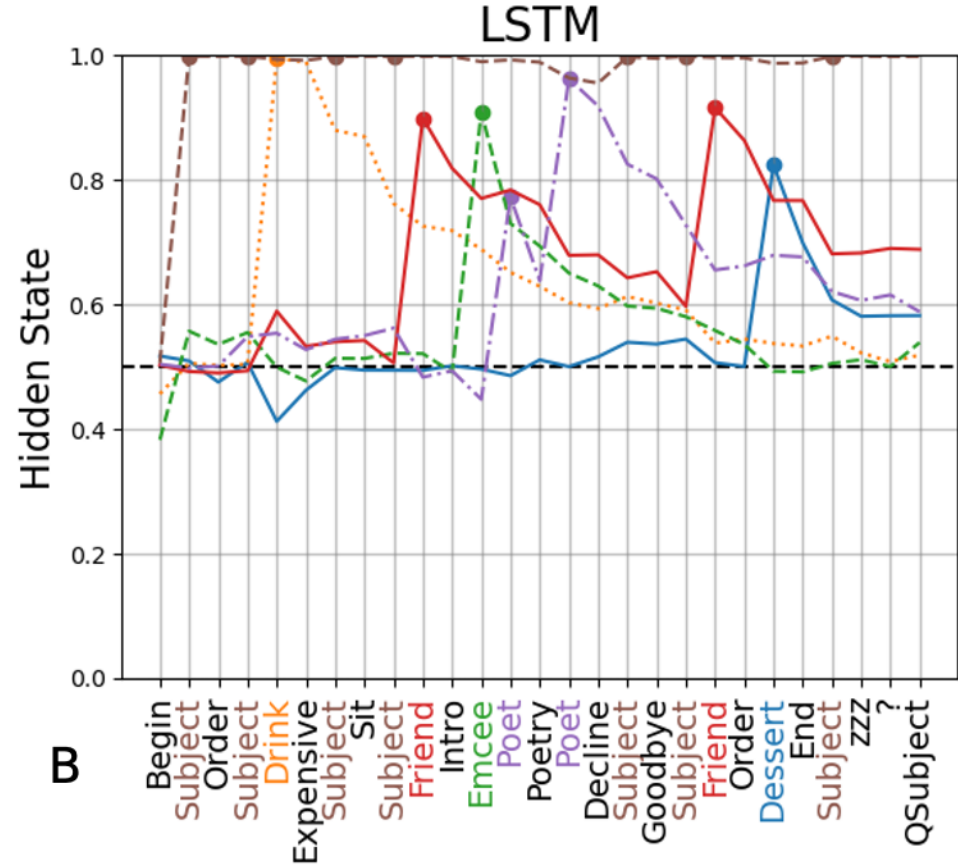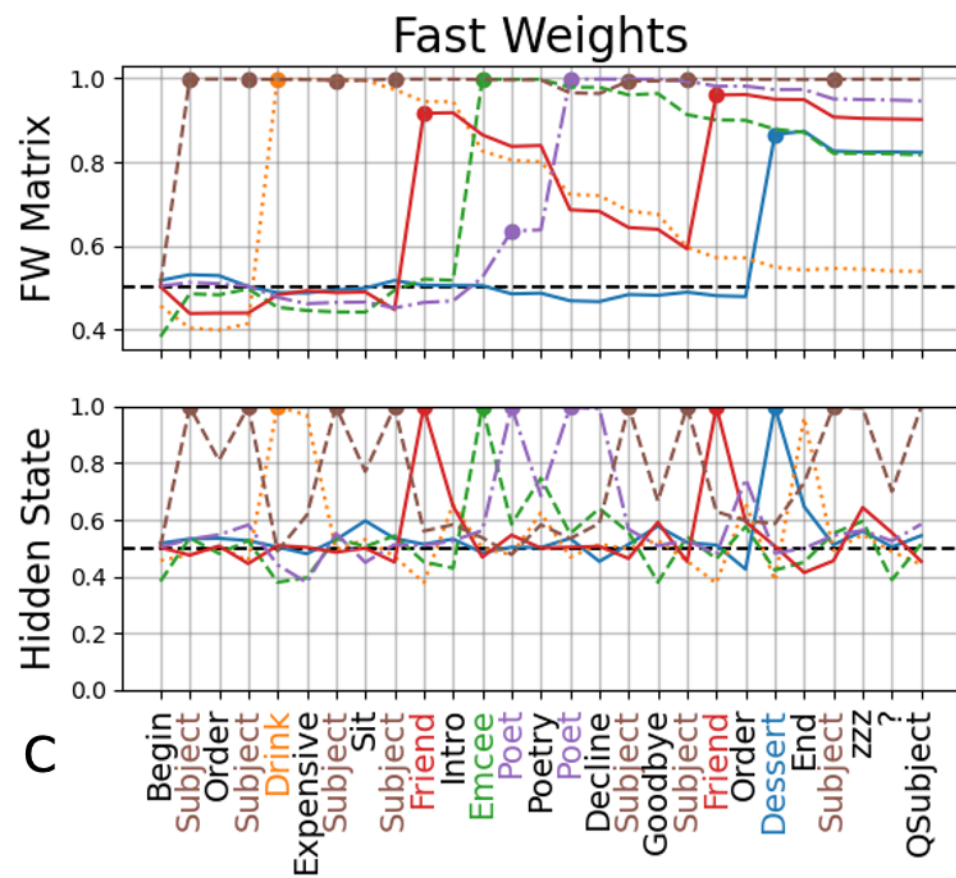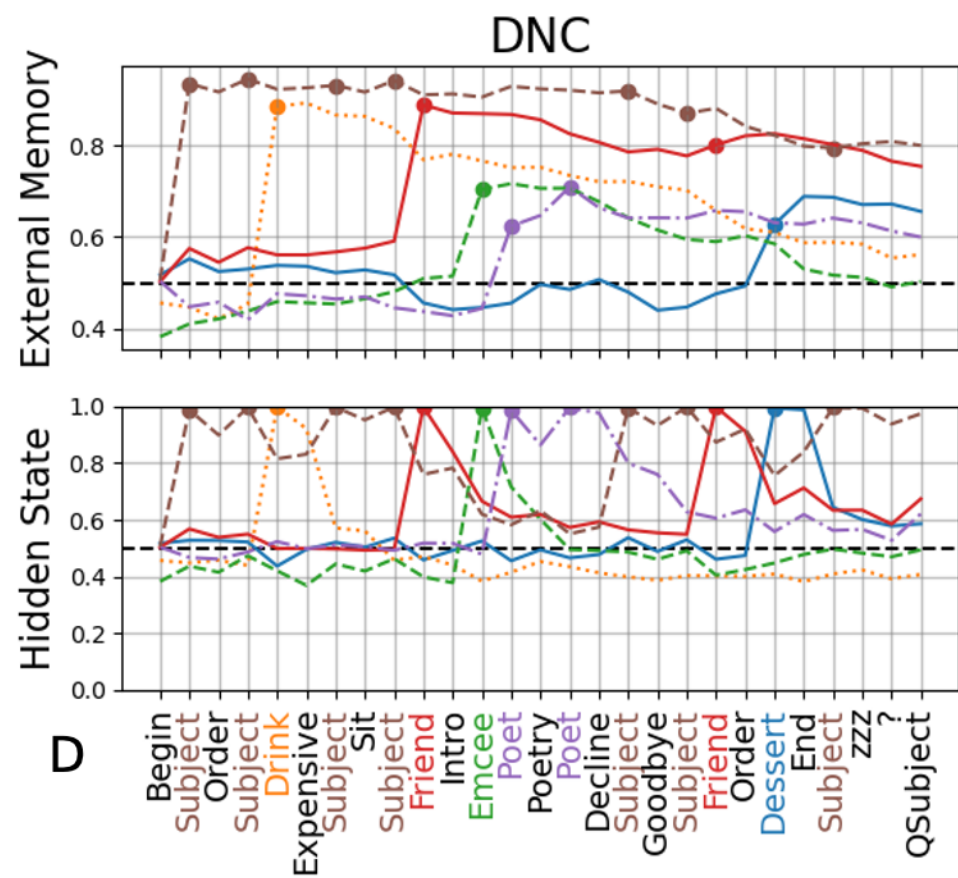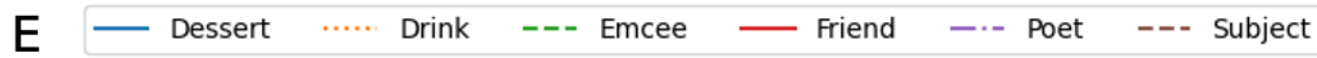

Supplement: Supplemental Information 5 [file peerj-09-11046-s005.pdf]

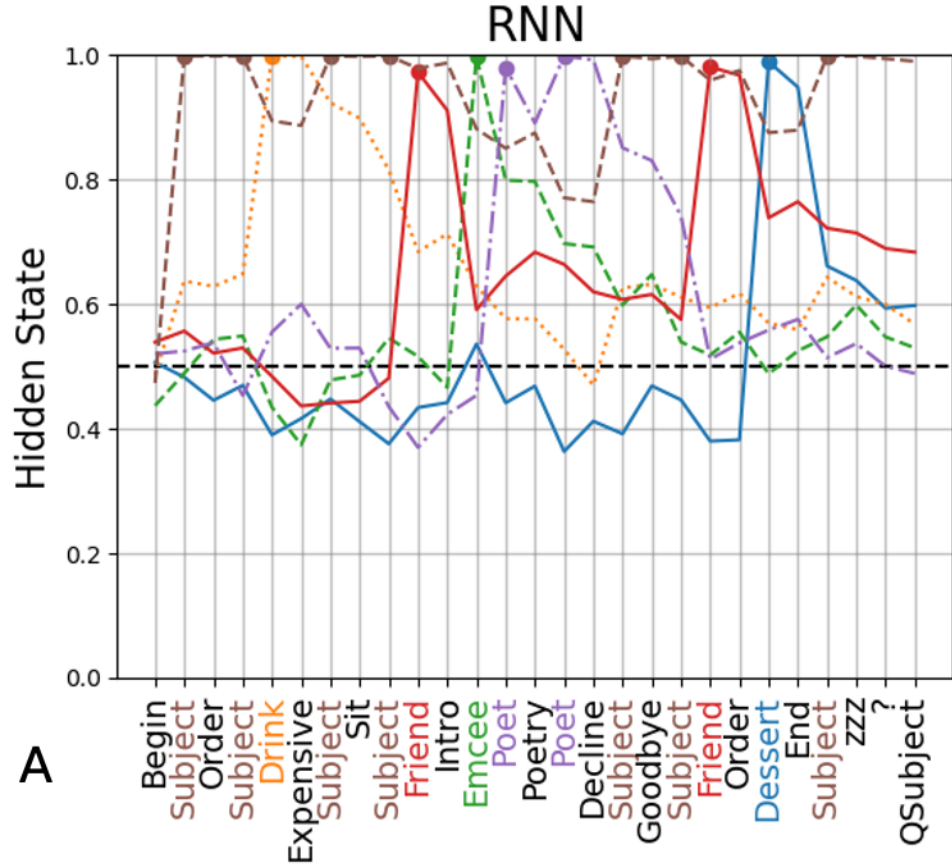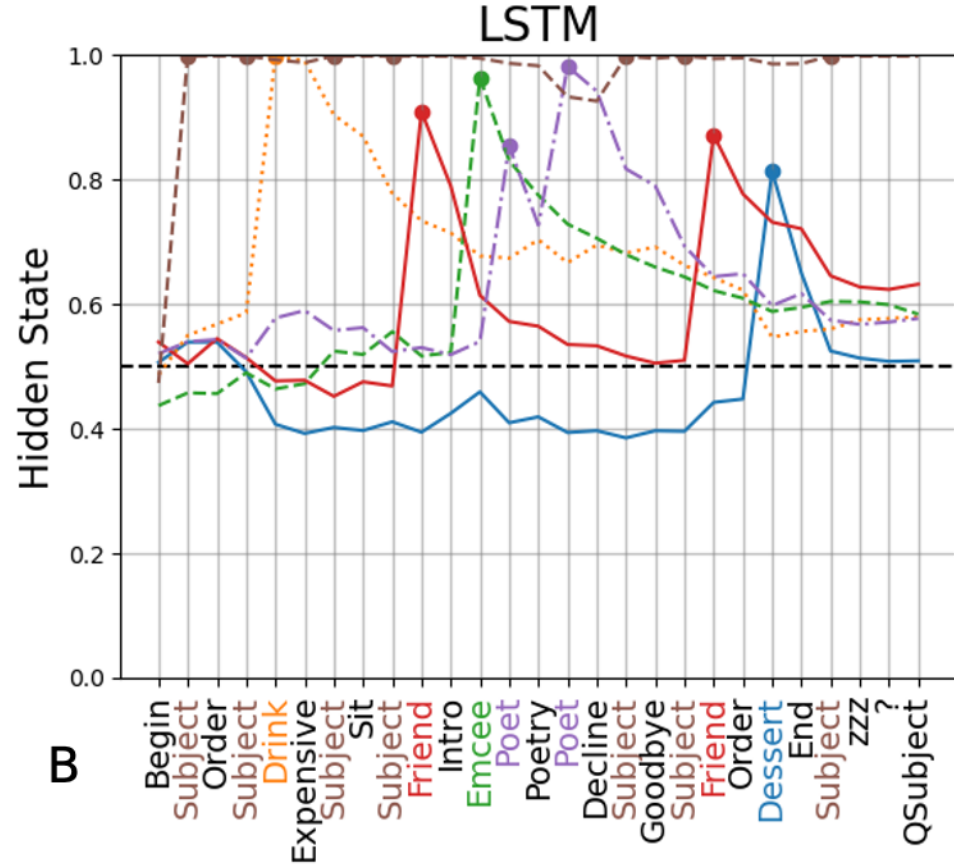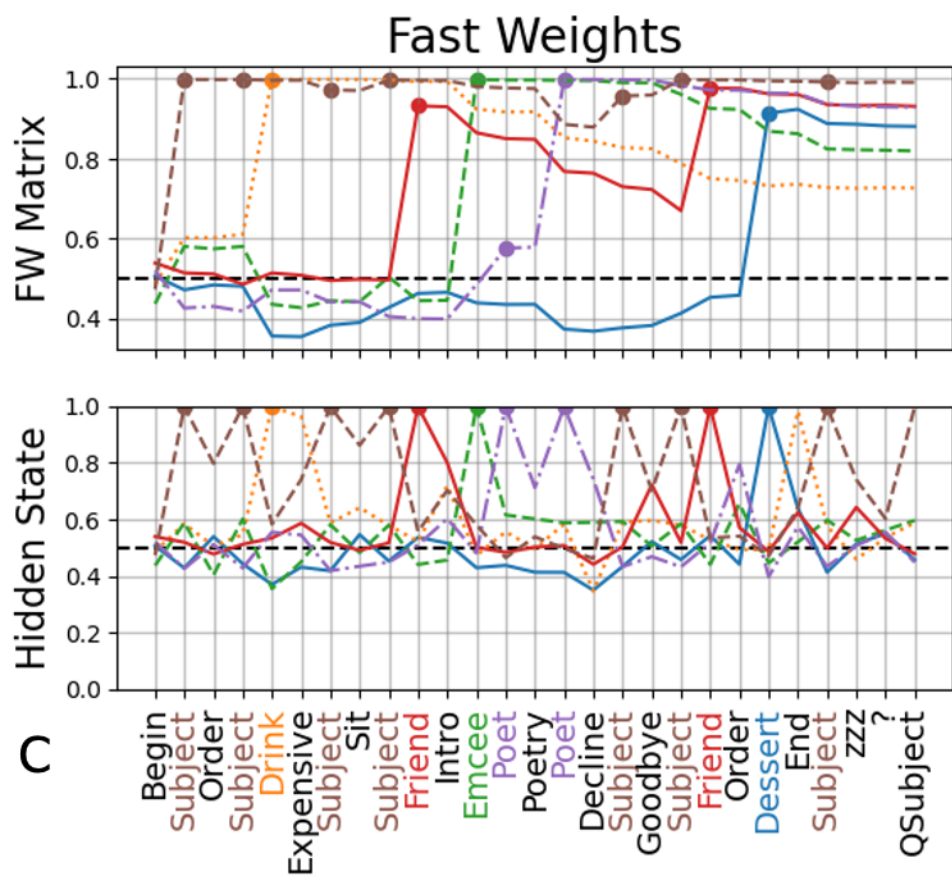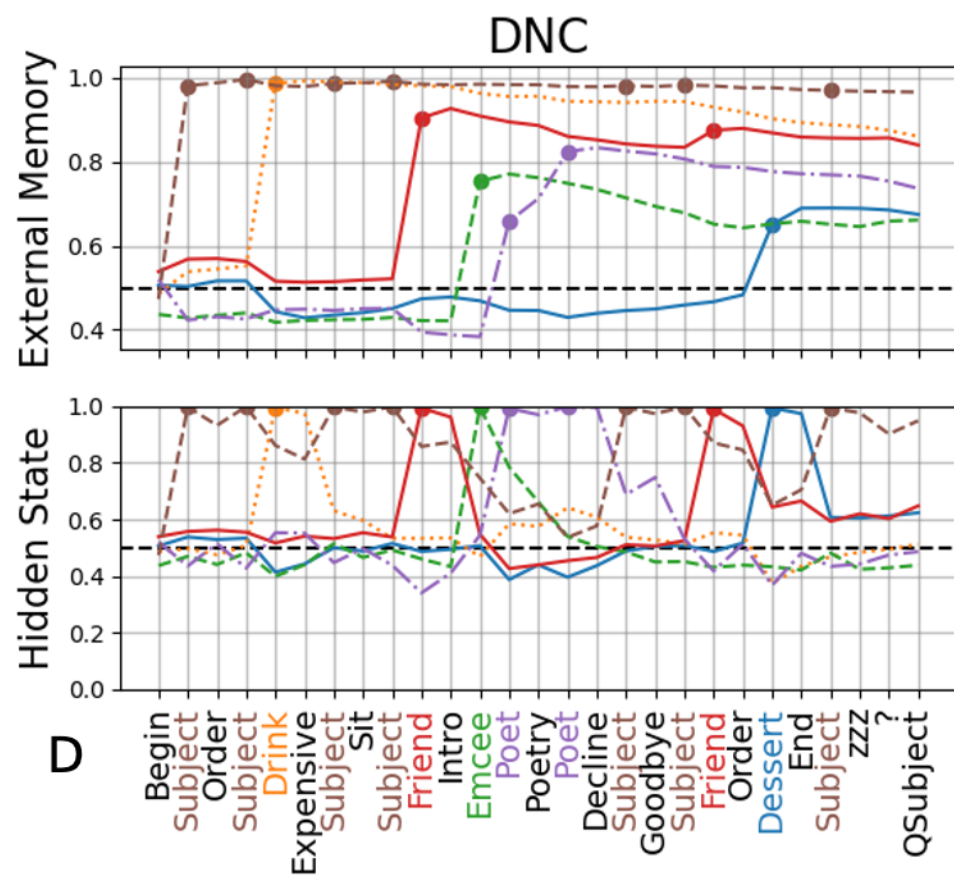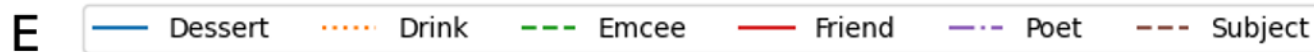

Supplement: Supplemental Information 6 [file peerj-09-11046-s006.pdf]

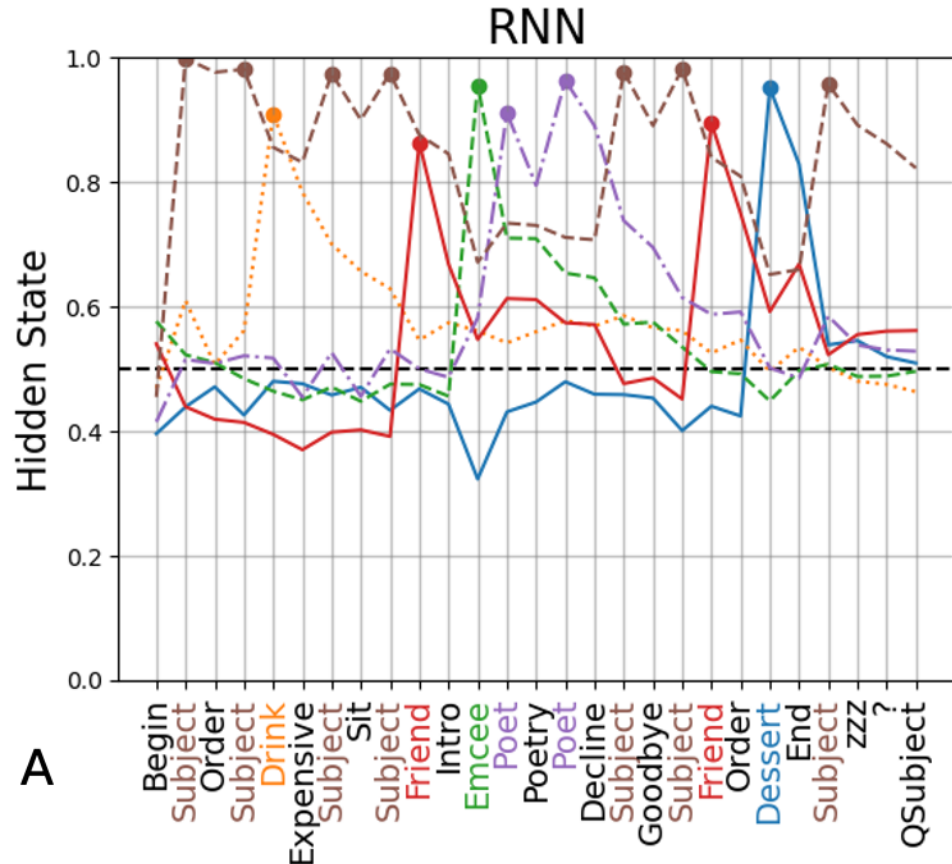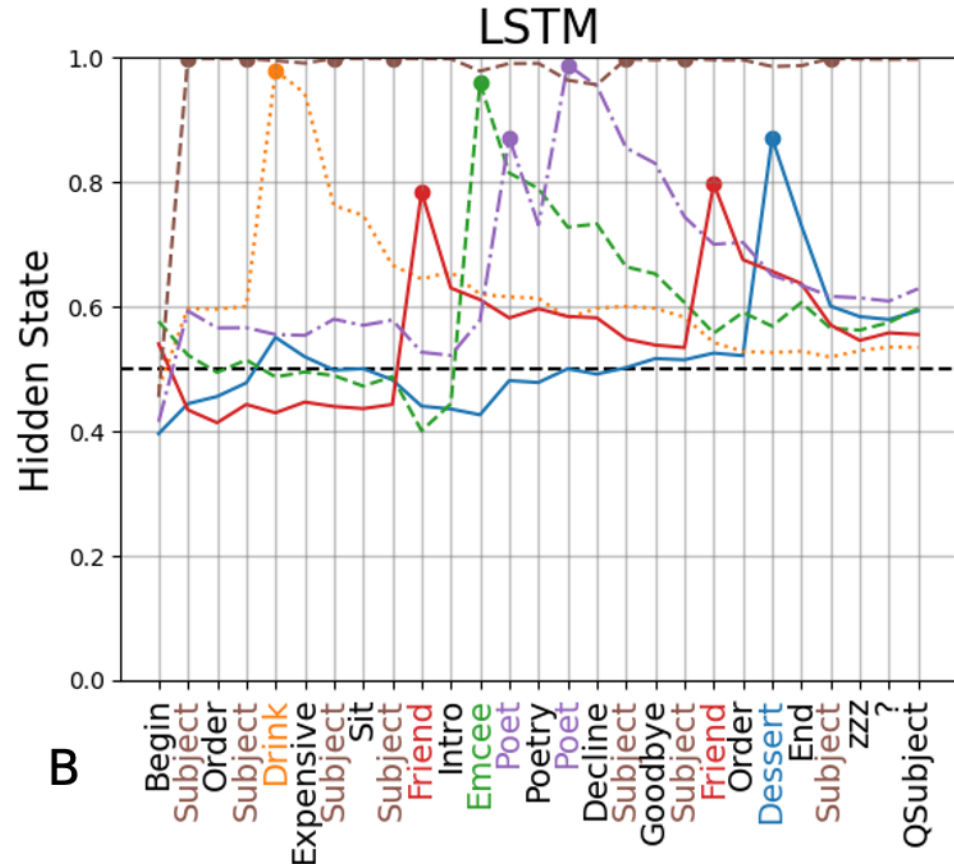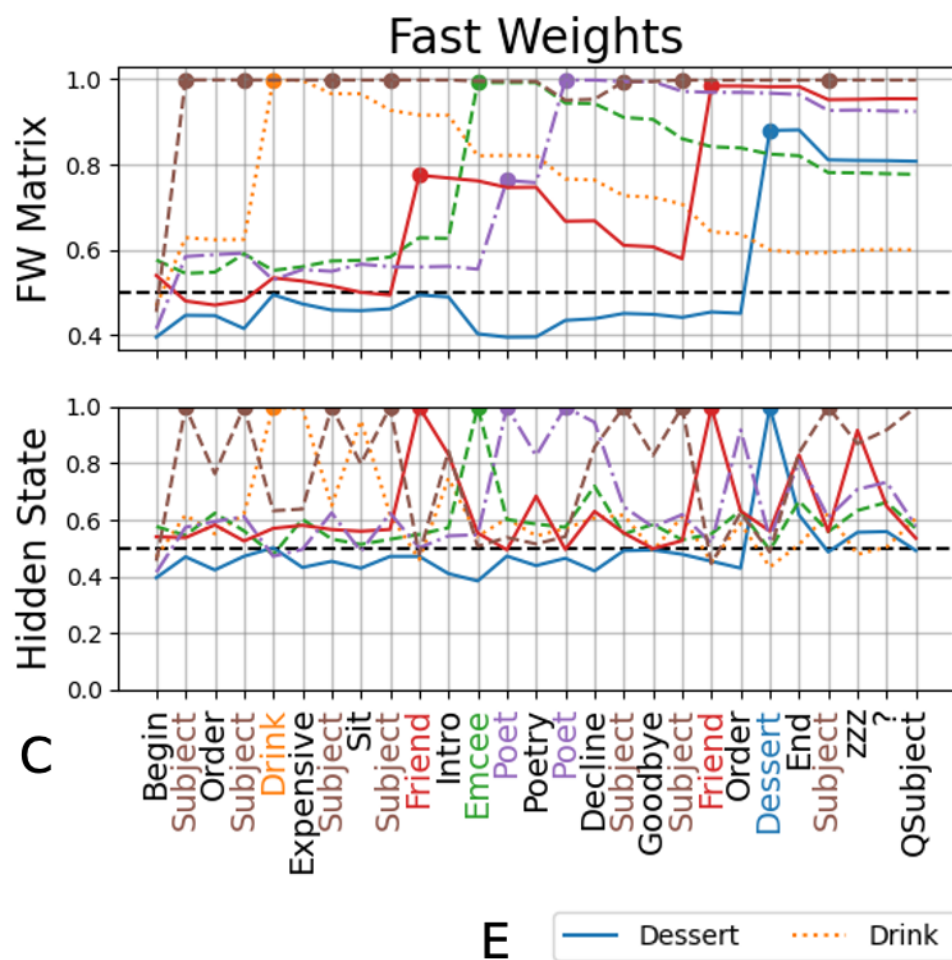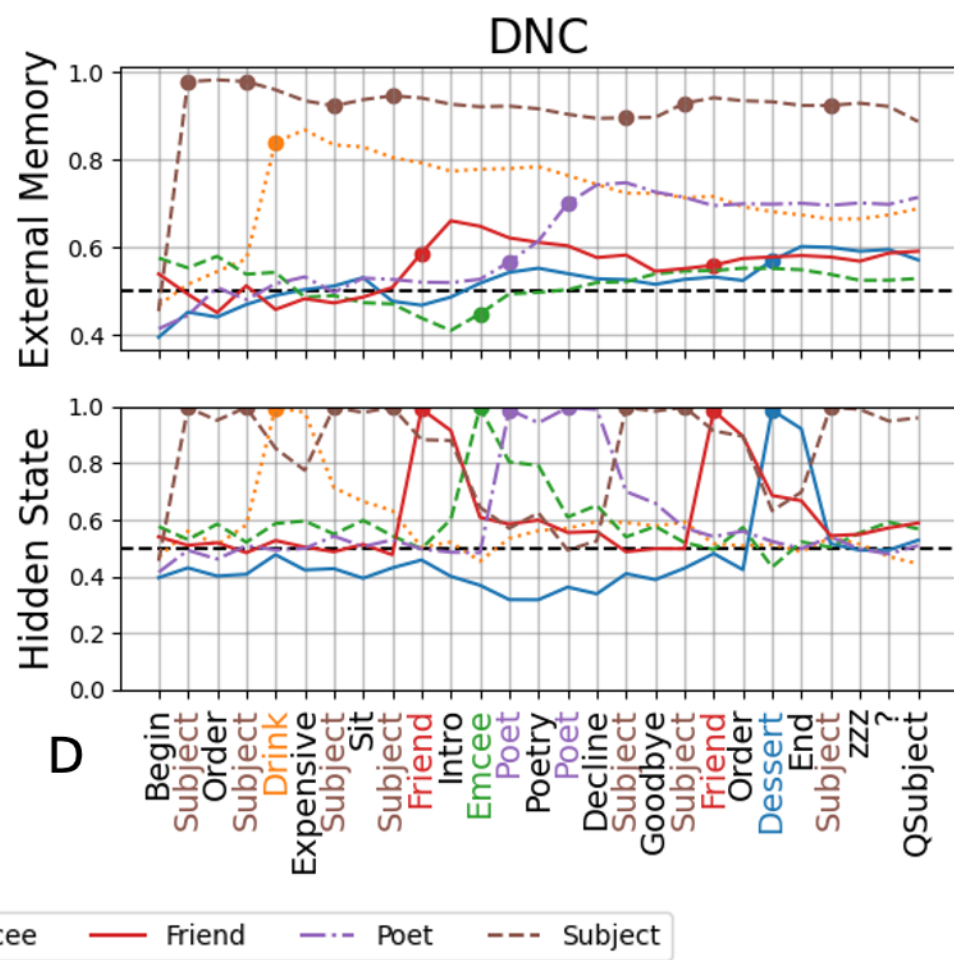

Supplement: Supplemental Information 7 [file peerj-09-11046-s007.pdf]
